# Immune Augmentation of Single Contact Hepatitis B Vaccine by Using PLGA Microspheres as an Adjuvant

**DOI:** 10.4103/0250-474X.44599

**Published:** 2008

**Authors:** S. M. Sivakumar, N. Sukumaran, R. Murugesan, T. S. Shanmugarajan, J. Anbu, L. Sivakumar, B. Anilbabu, G. Srinivasarao, V. Ravichandran

**Affiliations:** Vel’s College of Pharmacy, Pallavaram, Chennai-600 117, India; 1 Sri Paramakalyani Centre for Environmental Sciences, Maonmaniam Sundaranar University, Alwarkurichi-627 412, India; 2 Sophisticated Analytical Instrument Facility, Indian Institute of Technology, Chennai-600 036, India

**Keywords:** Immune augmentation, adjuvant, PLGA microspheres, hepatitis B vaccine delivery

## Abstract

The present study was aimed to replace the alum type adjuvant for hepatitis B vaccine. The hepatitis B vaccine was encapsulated in poly (DL-lactide-co-glycolide) microspheres by solvent evaporation technique. The formulated microspheres were characterized in terms of morphology, particle size analysis, *in vitro* release study and *in vivo* immune response in male Wistar rats. The FT IR spectrum illustrates the characteristics bands of poly (DL-lactide-co-glycolide) microspheres and hepatitis B vaccine at 1750 cm^-1^ and 1650 cm^-1^, respectively. The hepatitis B vaccine loaded poly (DL-lactide-co-glycolide) microspheres were able to release antigens till day 42. Significant enhancement of specific antibodies to HBsAg was produced till day 90 after a single administration of HBsAg encapsulated poly (DL-lactide-co-glycolide) microspheres. However, the conventional alum adsorbed hepatitis B vaccine was not found to produce any significant specific antibody levels till day 90 after a single dose. The results showed that poly (DL-lactide-co-glycolide) microspheres show potential as an adjuvant for hepatitis B vaccine.

Hepatitis B is an acute systemic infection, which is a major health problem all over the world. Hepatitis B virus infection can lead to fulminant hepatic failure that threatens the life of affected patients^1^. Moreover, the threat of infection may vary from country to country and approximately 30% of world’s population has serological evidence of hepatitis B infection^2^. There is no specific treatment even though certain drugs like interferon α and lamivudine are claimed to cure hepatitis B but the magnitude of these drugs also varies and responds to certain patients only^3^,^4^. Therefore, vaccination is the only way to alter the disease prevalence. Adjuvants have been used in the vaccine formulations to augment the immune response. Currently, alum is the only adjuvant approved for human use and plays a major role in vaccine delivery. However, alum is not an universal adjuvant as it is not suitable for small peptides. The conventional hepatitis B immunization schedules lead to dropout due to economical crisis, social and cultural conflict to the use of syringes. Therefore, it is necessary to find out an alternative vaccine delivery system to elicit a long lasting immune response and must give full protection through out the life after a single administration of vaccine. Besides, the immune response declines with increasing age and repeated immunization may result in seroconversions.

The development of single administration hepatitis B vaccine based on biodegradable polymers is a very important progress towards the betterment of human health care. Several studies have revealed that the polymer PLGA possesses valuable properties to improve the immune response of vaccines and assures to use as vaccine carrier^5^. The present study investigated the suitability of PLGA polymeric microspheres as an adjuvant for hepatitis B vaccine.

## MATERIALS AND METHODS

Poly (DL-lactide-co-glycolide) (PLGA) copolymer, Polyvinyl alcohol (PVA, 87-89% hydrolyzed) and Dichloromethane (reagent grade) were purchased from Sigma Aldrich, USA. The hepatitis B vaccine was obtained as a free sample from Serum Institute of India, Pune and all other chemicals were of reagent grade. In this research work JEOL JSM 5300 scanning electron microscope was used to observe morphology and surface characteristics. Spectrum one FT IR spectrophotometer (Perkin-Elmer) was used in the qualitative analysis of hepatitis B vaccine loaded PLGA microspheres. The immunogenecity studies were carried out in healthy Wistar rats. The animals were maintained and handled according to the standard guidelines of Institutional animal ethical committee 290/CPCSEA/2006.

Briefly, the vaccine encapsulated microspheres were prepared by solvent evaporation technique. The aqueous solution of HBsAg was dispersed in to organic phase consisting of polymer dissolved in dichloromethane and 0.05% (w/v) of Tween 80 was added. The emulsion so formed was sonicated at 15000 rpm for 20s and dispersed in 1% (v/v) glutaraldehyde in a magnetic stirrer for 10 min to stabilize the emulsion. The resulting emulsion was diluted in 400 ml of 1% (v/v) polyvinyl alcohol solution. The system was maintained under magnetic stirrer for 6 h to allow the solvent evaporation. The microspheres were collected by centrifugation at 10,000 rpm for 20 min. Microspheres were suspended in 10 ml of distilled water and filtered through a membrane filter and dried in a vacuum dessicator. The hepatitis B vaccine encapsulated microspheres thus obtained were pooled and stored in a refrigerator. Samples for SEM were mounted onto metal stubs and coated with gold palladium alloy to a thickness of 200-300 A°. The average diameter of hepatitis B vaccine loaded microspheres was found out by optical microscope in which stage micrometer was employed. The magnitude of loading of hepatitis B vaccine in microspheres was performed by a method established method by van der Lubben *et al*^6^. The loading capacity was found by mixing 100 mg of vaccine encapsulated microspheres with phosphate buffer saline (pH 7.3) under shaking at room temperature and kept for 3 h. The suspension was centrifuged at 3000 rpm for 30 minutes to remove free unloaded vaccine. The loading degree was determined by quantifying the non bound hepatitis B vaccine in the supernatant with the Lowry’s protein assay method. The loading of microspheres was calculated using the following formula, total amount of hepatitis B vaccine incorporated-free hepatitis B vaccine/total amount of hepatitis B vaccine. After loading of vaccine, the interaction between PLGA microparticle and hepatitis B vaccine was carried out by using FT IR technique at the range 450-4500 cm^-1^. The water uptake of hepatitis B vaccine encapsulated was performed by the method reported by Alonso *et al*^7^. Preformed microspheres (100 mg) were suspended in 20 ml of PBS (pH 7.4) containing 0.05% (w/v) of Tween 80 at room temperature for 24 h to measure water uptake. Microspheres were filtered, collected, weighed immediately (W_1_) and dried. After drying the microspheres were weighed (W_2_). The percentage water uptake (W) of microspheres was calculated by applying the following formula W= (W_1_-W_2_ )/W_2_×100. The stability of vaccine loaded and unloaded PLGA microspheres were determined over a period of 4 w. The microspheres were exposed at 4°, room temperature and at 50°.

Fifty milligrams of hepatitis B vaccine encapsulated microspheres were suspended in 100 ml of phosphate buffer saline pH 7.4 containing 0.2% (w/v) Tween 80 and 0.1% (w/v) of sodium azide in a 100 ml of conical flask and incubated at 37° by keeping the flask in a shaker cum incubator. The shaker was adjusted to 80 horizontal strokes/min. At pre determined time intervals that include 0, 1, 2, 4, 8, 12, 21, 28, 35, 42, 49 and 56 d, 5 ml of vaccine releasing solution was removed and fresh medium was replaced. The samples were centrifuged at 4000 × g for 15 min and the supernatant solution was analysed by enzyme immunoassay using Immulite 2000 automated analyzer for HBsAg (Diagnostic Products Corporation, Los Angles, CA, USA). The magnitude of HBsAg in the samples was calculated by extrapolation on the standard curve.

Two Groups (6 per group) of male Wistar rats were immunized intramuscularly on the thigh region with single dose of hepatitis B vaccine encapsulated PLGA microspheres and standard alum adsorbed hepatitis B vaccine, respectively. The blood samples were collected from immunized Wistar rats by retro orbital plexus using capillary tube. The collected blood samples were pooled and serum was separated by centrifugation and stored at -20°. The potency of hepatitis B vaccine encapsulated PLGA microspheres was determined by measuring specific antibodies through enzyme immunoassay using Immulite 2000 automated analyzer for antiHBs (Diagnostic Products Corporation, Los Angles, CA, USA). The immunoglobulin titers were performed using Orion Diagnostica Turbox IgG and IgM assay kits.

**Fig. 1:**
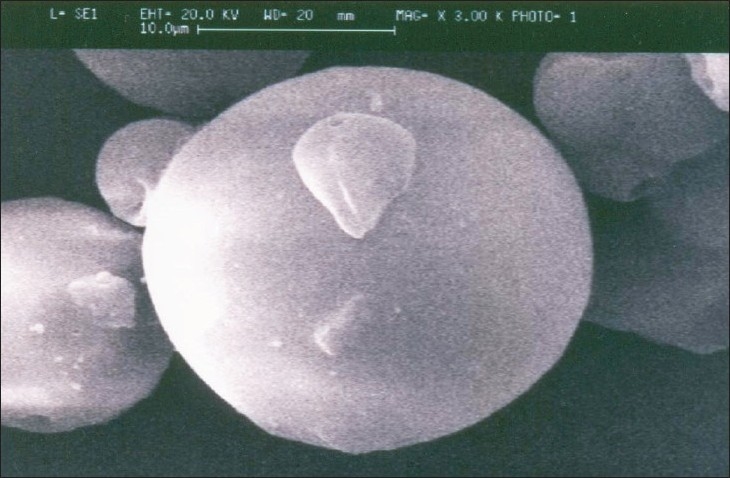
Scanning electron micrograph of hepatitis B vaccine encapsulated PLGA microspheres

Vaccines are considered as the most successful medical interventions against infectious disease. The World Health Organization aims to develop more economical and effective vaccine against various diseases. Accordingly, the development of more efficient and safe vaccine delivery system requiring single administration to obtain long lasting immune response is therapeutically and economically more important. Therefore, traditionally, adjuvants have been used in the vaccine formulation to improve vaccine efficacy. During the last 70 y, many adjuvant formulations have been developed and a few of these have been evaluated in clinical trials. However, most of these were not accepted for routine vaccines, mainly due to their toxicity and side effects. The use of alum type adjuvant for immunization, however, has several disadvantages because it induces inflammation and stimulates the local production of granuloma. In addition alum is not a universal adjuvant as it is not suitable for small peptides, recombinant proteins and alum cannot be frozen or lyophilized. Conventional alum type vaccines require multiple recall injections at appropriately timed intervals in order to achieve long lasting and optimal immune response. However, it is very difficult, especially in developing countries, to maintain a high reimmunization rate in the case of multiple administration immunization programs. Therefore, development of more efficient and safe adjuvant/vaccine delivery systems requiring single administration to obtain high and long lasting immune responses is of primary importance. During past two decades considerable efforts have been made to utilize the biodegradable polymers as an adjuvant for vaccine delivery. Controlled release of antigens from polymer microparticles has been of particular interest to those interested in the development of vaccines, which would be effective in a single contact immunization^8^. Recently, the concept of microparticulate technology is focused towards hepatitis B vaccine. In this research work, the suitability of PLGA microspheres produced by solvent evaporation technique to entrap, conserve the physicochemical properties and antigenicity of hepatitis B vaccine was found. The hepatitis B vaccine loaded PLGA microspheres were homogenous and having smooth surfaces (fig. 1). The diameter of the microspheres was 10 to 50 μm. The loading capacity was found to be 70% and 30% of hepatitis B vaccine was lost during micro encapsulation process. The loading of vaccine in to the microspheres and the size of the microspheres were greatly influenced by stabilizing agent concentration, polymer concentration and sonication process. The morphology of hepatitis B vaccine encapsulated PLGA microspheres was not changed at 4° and at room temperature but not at 50°. The water uptake of vaccine loaded microspheres after 24 h incubation led to release of antigen from microspheres. It is presumed that water penetrates in to the microparticles, dissolves the cross linking agent and causes osmotic pressure. Accordingly, the pore size of microparticles increased and thus the vaccine was released. A similar behavior was observed for TT encapsulated PLGA microspheres^6^. In FT IR spectrum, the bands at 1650 and 1750 cm^-1^ clearly indicates the presence carbonyl group, saturated aliphatic ester and primary amine, which confirms the characteristic bands of hepatitis B vaccine and PLGA microspheres.

**Fig. 2:**
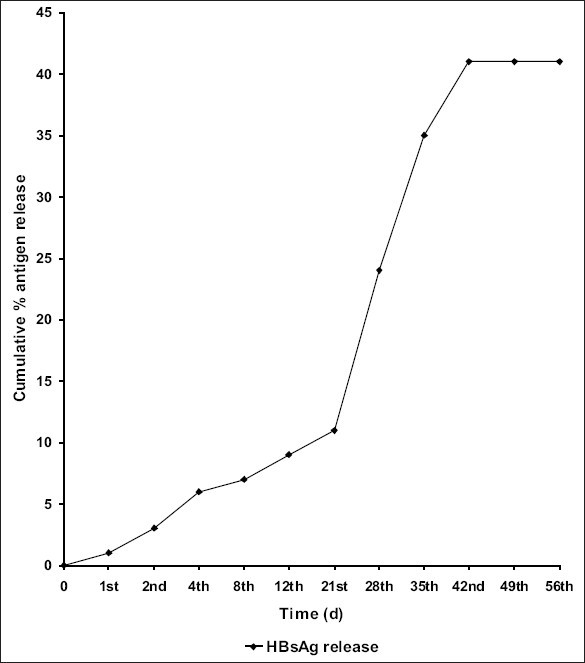
*In vitro* release study of hepatitis B vaccine encapsulated PLGA microspheres

The *in vitro* release profile (fig. 2) showed that the entrapped antigen was released gradually from d 1 and the peak antigen was released on d 42. However, the percentage of total protein and antigenic active protein released from microspheres were entirely different. It clearly indicates that there might be some degradation of hepatitis B vaccine during micro encapsulation process. Therefore, large amount of hepatitis B vaccine was incorporated during micro encapsulation process, which increases the loading capacity but unfortunately it results in particle aggregation. With respect to the release of antigen from PLGA microspheres *in vitro*, the *in vivo* results also confirmed the release of antigen. The immune response is a complex mechanism is triggered when an antigen enter in to the body. The results tabulated in Table 1 represents a comparative studies on specific immunoglobulin, IgG and IgM levels on days 45 and 90. Dunnet multiple comparison test was performed to compare the immunoglobulin levels of test samples with control values. From the results it is very obvious that the antiHBs response on days 45 and 90 after a single step of immunization with hepatitis B vaccine encapsulated PLGA microspheres was greater when compared to conventional alum adsorbed hepatitis B vaccine. In this research work it has been determined that the IgG and IgM levels were elevated even on d 90.

**TABLE 1:** A COMPARITIVE STUDY ON SERUM ANTIHBS, IgG AND IgM LEVELS

Groups	Anti HBs ( IU/l)		IgG (mg/dl)		IgG (mg/dl)	
			
	45^th^ d	90^th^ d	45^th^ d	90^th^ d	45^th^ d	90^th^ d
Group 1	10.2±1.6	11±2.3	791.6±2.4	793.3±1.3	82.2±1.6	75.6±2.1
Group 2	10.1±1.3	8.1±0.8	791.6±1.7	789.6±2.3	83.1±1.2	77.1±1.4
Group 3	-	-	705±7.6	708.3±7.9	62.6±0.6	61.8±0.5

Group 1 animals received hepatitis B vaccine encapsulated PLGA microspheres and Group 2 animals received conventional alum adsorbed hepatitis B vaccine. Group 3 is the control animals did not receive any vaccine formulation. Each value is a mean of 6 batches with a standard deviation and all the values were extremely significant (P< 0.01).

The size of the microspheres also dictates the immune response of vaccine and after an intramuscular injection, the PLGA microspheres ranging in diameter of 10μm are readily phagocytosed by macrophages. The particle size more than 10μm in diameter would remain as a depot at the site of injection and need to undergo degradation before phagocytosis can occur. This might provide the sustained release of hepatitis B vaccine to elicit a long lasting immune response. The required amount of anti HBs level for protection is 10 IU/l after vaccination. In this study, interestingly significant anti HBs level was observed even up to d 90 after a single dose of hepatitis B vaccine encapsulated PLGA microspheres. However, the conventional alum adsorbed hepatitis B vaccine was not produced significant antibodies level up to d 90 after a single dose of immunization. Therefore, it is clearly demonstrated that the total immune system was actively participated to elevate acquired immunity against HBsAg encapsulated PLGA microspheres. The present study clearly indicates that the PLGA microparticle system can release HBsAg gradually but steadily. Further studies are needed to determine the optimum dosage and in order to overcome the lacunae that we experienced in this research work.
